# A WeChat-based, expert knowledge-constrained artificial intelligence health education intervention for patients undergoing gastrointestinal surgery: protocol for a randomized controlled trial

**DOI:** 10.3389/fpubh.2026.1862627

**Published:** 2026-09-30

**Authors:** Li Liu, Xu Du, Guangli Yang, Shuyao Xiang, Hua Guo, Lu Huang, Yun-Hua Li, Hongtao Yu

**Affiliations:** 1 Department of Medical Affairs, The Third People's Hospital of Chengdu, Chengdu, China; 2 Department of Nursing, The Fourth People's Hospital of Chengdu, Chengdu, China; 3 Department of Medical Affairs, Chengdu Women's and Children's Central Hospital, Chengdu, China; 4 Department of Quality Evaluation and Medical Record Management, The Third People's Hospital of Chengdu, Chengdu, China; 5 Department of Gastroenterology, The Fourth People's Hospital of Zigong, Zigong, China; 6 School of Education, Chengdu College of Arts and Sciences, Chengdu, China; 7 Hospital Administration Office, The Third People's Hospital of Chengdu, Chengdu, China

**Keywords:** artificial intelligence, gastrointestinal surgery, health education, perioperative care, randomized controlled trial

## Abstract

**Background:**

Perioperative health education is essential for patients undergoing gastrointestinal surgery, but conventional approaches are often limited by time, workload, continuity, and personalization. Artificial intelligence (AI) may offer a more accessible and interactive approach to patient education. This trial aims to evaluate whether a WeChat-based, expert knowledge-constrained AI health education system can improve anxiety and other patient-reported outcomes in patients undergoing gastrointestinal surgery.

**Methods:**

This is a single-center, open-label, parallel-group randomized controlled trial with a 1:1 allocation ratio. A total of 120 eligible patients will be recruited from the Department of Gastrointestinal Surgery at Chengdu Third People’s Hospital and randomly assigned to either an intervention group or a control group. The control group will receive usual perioperative care and conventional health education, while the intervention group will additionally receive a WeChat-based AI-supported health education intervention for 3 months. The primary outcome is anxiety measured by the Hospital Anxiety and Depression Scale-Anxiety subscale. Secondary outcomes include depression, perceived social support, self-efficacy, health-related quality of life, and eHealth literacy. Outcomes will be assessed at baseline, discharge, 3 months, and 6 months. Analyses will primarily follow the intention-to-treat principle.

**Ethics and dissemination:**

This study is a low-risk behavioral intervention trial. Study findings will be disseminated through peer-reviewed publications and other academic channels. Ethical approval for the study was obtained from the Medical Ethics Committee of Chengdu Third People’s Hospital (Ethics No: 2026-S-69). The clinical trial is registered under ChiCTR2600122411, with registration completed on April 2026.

**Clinical trial registration:**

https://www.chictr.org.cn/, identifier ChiCTR2600122411.

## 1 Introduction

Gastrointestinal diseases that require surgical treatment, especially gastric cancer and colorectal cancer, remain an important component of the global cancer burden and surgical treatment (1–3). Patients undergoing gastrointestinal surgery often face complex perioperative challenges, including anxiety about the surgical process, postoperative recovery, symptom management, diet, and potential complications (4, 5). These challenges are often accompanied by psychological stress, especially anxiety, which may have adverse effects on treatment experience, treatment compliance, and rehabilitation (6–8). Therefore, perioperative patient education is an important component of surgical nursing, and existing evidence suggests that well-designed educational interventions can improve patient experience and help achieve better postoperative outcomes (9–11). This clinical background provides a strong basis for developing more accessible and effective health education strategies for gastrointestinal surgery patients.

Although perioperative patient education is recommended as part of “accelerated rehabilitation surgical nursing,” the evidence base in the field of abdominal surgery is still inconsistent, with significant differences in content, timing, implementation methods, and outcome evaluation (5, 9, 12). In addition, surgical patients often report that their educational expectations during the perioperative period have not been fully met, indicating a significant gap between the information they need and the support they actually receive (13–15). Recent studies using mixed research methods further suggest that effective perioperative education should be a two-way process involving active patient participation and continuous feedback, rather than relying solely on one-way information transmission (15, 16). Digital approaches may help address some of these limitations, but perioperative qualitative studies show that patients want support that is accessible, trusted, personalized, and available across both pre- and postoperative phases (17, 18). Meanwhile, systematic reviews of perioperative chatbots conclude that the field is promising but still supported by limited and heterogeneous evidence (17, 19).

Artificial intelligence (AI), especially conversational intelligent assistants based on Large Language Models (LLMs), provides a potential way to overcome the limitations of traditional perioperative education by offering timely, convenient, and highly interactive support to patients. Recent reviews have shown that LLM based tools can improve patient education by enhancing information accessibility, personalization, and engagement (20, 21). Research on perioperative chatbots shows that patients generally have a high acceptance rate, and this technology has potential benefits in education and experience (19, 22). However, there are still issues with directly using unrestricted universal LLM in medical settings, as its response may be inconsistent, lack transparency, and prone to hallucinations or clinically unsafe content (23, 24). Therefore, emerging literature emphasizes that patient oriented medical artificial intelligence should be developed within a framework that prioritizes safety, factual basis, and regulatory accountability (24–26). In this context, a more appropriate approach for gastrointestinal surgery education is to combine conversational artificial intelligence interfaces with expert reviewed knowledge bases, ensuring that response content is limited to validated educational content rather than unlimited text generation. This theoretical basis forms the foundation of this study.

Despite rapidly increasing interest in AI-supported patient education, the current evidence base for perioperative care remains limited, and evidence specific to gastrointestinal surgery is even more sparse. Recent systematic reviews suggest that perioperative chatbots are generally acceptable to patients and may improve knowledge, satisfaction, and aspects of perioperative experience (19). However, the available studies are heterogeneous in design, intervention content, and outcome measurement (19). More broadly, reviews of LLMs in patient education and patient care have highlighted their potential to improve accessibility, personalization, and engagement, while also emphasizing unresolved concerns regarding factual reliability, transparency, reproducibility, and safety (20, 23). Accordingly, there remains a need for randomized controlled studies in real-world gastrointestinal surgical settings, particularly for systems that constrain AI outputs through expert-curated knowledge bases or retrieval-augmented architectures rather than unrestricted text generation. The present study is informed by the Health Action Process Approach (HAPA) theory, which distinguishes a motivational phase, in which outcome expectancies and action self-efficacy contribute to intention formation, from a volitional phase, in which planning and self-regulatory processes support action initiation and maintenance (27, 28). Within this framework, AI-supported question answering and tailored educational content are expected to improve patient knowledge and strengthen outcome expectancies and self-efficacy, whereas reminder functions and repeated interaction may facilitate action planning, coping planning, and maintenance of perioperative self-management behaviors across the perioperative and post-discharge period. Against this background, the present trial aims to evaluate whether a WeChat-based AI health education system, constrained by an expert-reviewed knowledge base, can reduce anxiety and improve other patient-reported outcomes compared with usual health education in patients undergoing gastrointestinal surgery.

## 2 Methods: participants, interventions, and outcomes

### 2.1 Trial design and study setting

This study is a single-center, open-label, parallel-group randomized controlled trial with a 1:1 allocation ratio and a superiority framework. It will be conducted at Chengdu Third People’s Hospital, a tertiary general hospital in Chengdu, Sichuan Province, China, to compare an AI-supported health education intervention delivered via a WeChat public account with usual health education in patients undergoing gastrointestinal surgery. Participants will be recruited from the Department of Gastrointestinal Surgery and screened for eligibility according to the predefined inclusion and exclusion criteria (Figure 1). This protocol was developed and reported in accordance with the Standard Protocol Items: Recommendations for Interventional Trials (SPIRIT) 2013 statement.

**Figure 1:**
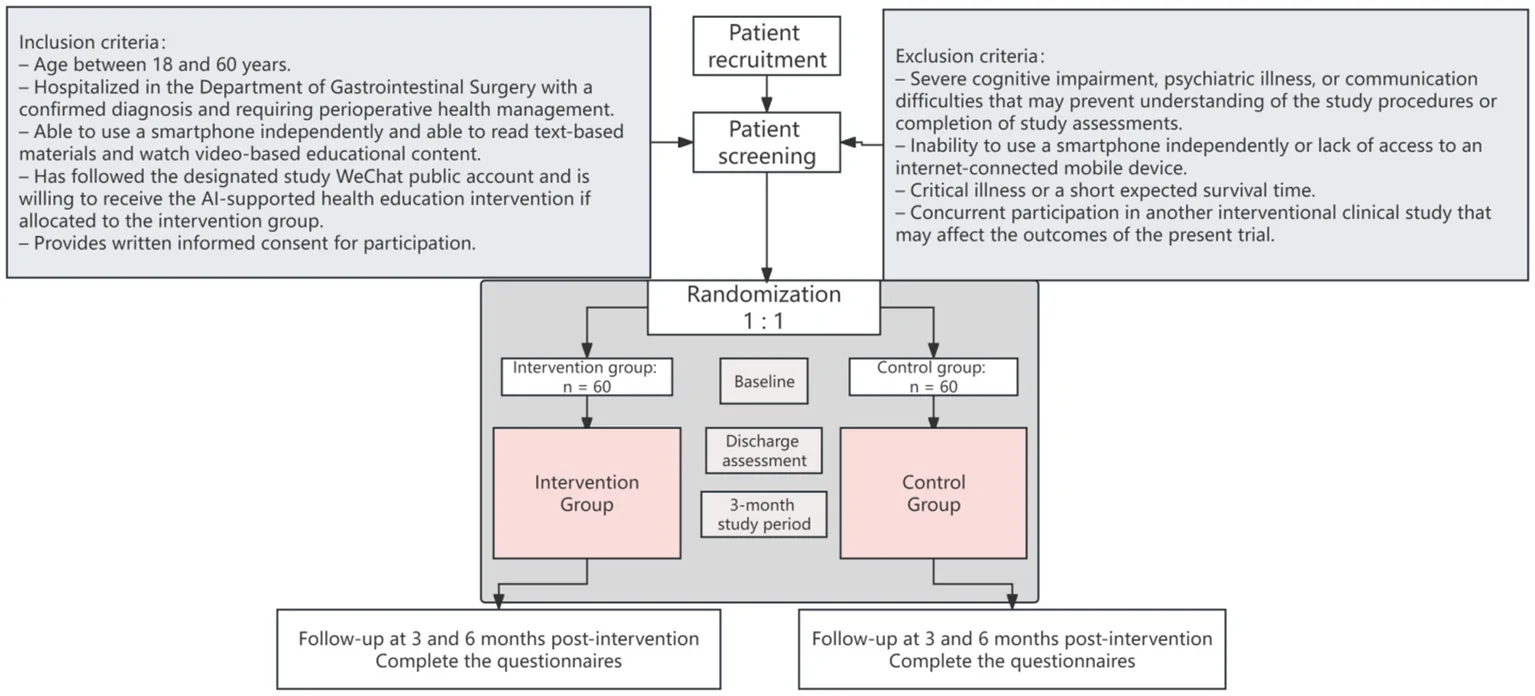
Flow chart of patient recruitment and study implementation.

### 2.2 Eligibility criteria

Patients will be screened for eligibility after hospital admission and before randomization. Participants must meet all of the following criteria to be eligible for the study:

#### 2.2.1 Inclusion criteria

- Age between 18 and 60 years.
- Hospitalized in the Department of Gastrointestinal Surgery with a confirmed diagnosis and requiring perioperative health management.
- Able to use a smartphone independently and able to read text-based materials and watch video-based educational content.
- Has followed the designated study WeChat public account and is willing to receive the AI-supported health education intervention if allocated to the intervention group.
- Provides written informed consent for participation.

#### 2.2.2 Exclusion criteria

Patients who meet any of the following criteria will not be eligible for the study:

- Severe cognitive impairment, psychiatric illness, or communication difficulties that may prevent understanding of the study procedures or completion of study assessments.
- Inability to use a smartphone independently or lack of access to an internet-connected mobile device.
- Critical illness or a short expected survival time.
- Concurrent participation in another interventional clinical study that may affect the outcomes of the present trial.

#### 2.2.3 Who will take informed consent?

Potentially eligible patients will be identified after admission to the Department of Gastrointestinal Surgery through review of medical records and confirmation with the attending clinical team. Patients who appear to meet the eligibility criteria will be approached before randomization by a registered research nurse who is a member of the study team and has received protocol-specific training in participant recruitment and informed consent procedures.

The research nurse will approach patients during hospitalization, after their initial clinical condition has been stabilized and at a time that does not interfere with routine clinical care. The study will be explained to potential participants in a quiet and private setting using both oral explanation and a written participant information sheet. The explanation will include the study purpose, intervention procedures, randomization, follow-up assessments, potential risks and benefits, confidentiality protections, voluntary participation, and the right to withdraw at any time without affecting routine medical care.

Patients will be given sufficient time to read the information sheet, consider participation, and ask questions. Written informed consent will be obtained by the registered research nurse before enrollment and before randomization. Only patients who have provided written informed consent will be included in the study.

Participants allocated to the intervention group will also be informed that their AI-system usage data, including access frequency, question categories, use of educational content, reading duration, and reminder use, will be collected for research and intervention fidelity assessment. They will be informed that these usage data will be anonymised or de-identified before analysis, will not contain directly personally identifiable information in the research dataset, and will be managed according to the study confidentiality and data protection procedures.

### 2.3 Interventions

#### 2.3.1 Intervention description

All participants will receive routine perioperative care in the Department of Gastrointestinal Surgery. Routine perioperative care includes standard medical and nursing management, perioperative assessment and treatment, postoperative monitoring, discharge guidance, and routine health education delivered by clinical staff.

Participants in the control group will receive routine perioperative care and conventional health education only. Conventional health education will be delivered by registered nurses with experience in gastrointestinal surgical nursing, and disease-specific explanations may be supplemented by surgeons when necessary. The routine education process will include: (1) one preoperative face-to-face education session after admission and before surgery, lasting approximately 20–30 min, covering disease-related knowledge, surgical preparation, fasting and drinking instructions, anesthesia-related precautions, respiratory training, early mobilization, pain management, and psychological support; (2) postoperative bedside education during hospitalization, generally lasting 10–15 min each time and provided according to the patient’s recovery status, covering postoperative diet, wound and drainage tube care, pain management, early mobilization, prevention of complications, and warning signs requiring medical attention; and (3) one discharge education session before discharge, lasting approximately 20–30 min, covering medication instructions, dietary management, wound or stoma care if applicable, rehabilitation recommendations, follow-up arrangements, and symptoms requiring timely medical consultation. Paper-based or routine departmental educational materials will be provided when appropriate. During the trial period, control group participants will not receive access to the AI-supported health education system, online question-answering services, semi-personalized content push, or reminder functions provided by the intervention platform.

Participants in the intervention group will receive routine perioperative care and conventional health education identical to those provided to the control group. In addition, they will receive access to a WeChat-based AI-supported health education system embedded in the department’s official WeChat account for 3 months (Figure 2). The AI-specific components of the intervention include natural language question answering based on an expert-reviewed knowledge base, retrieval-supported response generation through the Coze platform, semi-personalized educational content recommendation based on patient interaction data, and automated recording of usage patterns for intervention optimization. WeChat serves as the delivery interface through which the AI-supported functions are accessed. Before using the system, trained research nurses will provide a brief standardized instruction session, lasting approximately 10–15 min, to explain system access, basic functions, precautions for use, and the scope and limitations of the system. Participants will access the system through the department’s official WeChat account or a study-specific QR code.

**Figure 2:**
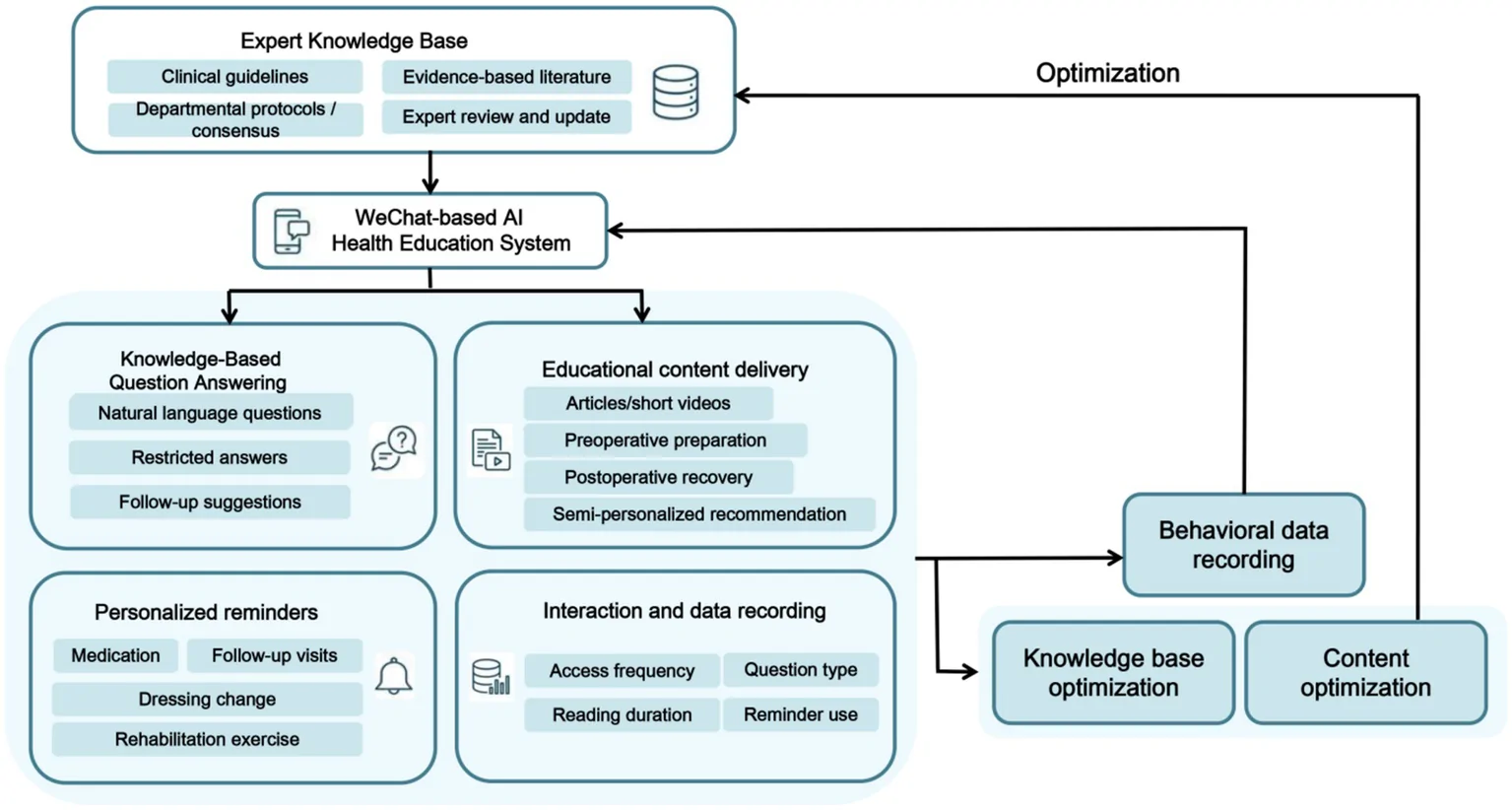
Intervention design and technical pathway of the WeChat-based AI health education system.

The system was jointly developed by gastrointestinal surgeons, nursing experts, and information technology personnel. Its core component is an expert knowledge-constrained health education knowledge base constructed from published clinical practice guidelines, evidence-based perioperative care literature, Enhanced Recovery After Surgery recommendations, clinical nutrition guidance for surgical patients, and internal departmental protocols and consensus materials (5, 12, 29). At the start of the trial, the knowledge base will consist of a structured, module-based set of expert-reviewed educational materials, frequently asked questions, and predefined response templates covering common gastrointestinal surgical diseases, perioperative preparation, postoperative recovery, dietary management, wound and stoma care, drainage tube care, complication identification, rehabilitation, medication and follow-up, and self-management after discharge. Each knowledge entry will include a topic title, applicable perioperative stage, target patient group or surgical context, source reference, key educational content, predefined safety prompts where applicable, and version information. The system is designed to respond to common health education questions related to perioperative preparation, postoperative recovery, symptom observation, diet, wound and drainage tube care, stoma care, rehabilitation, follow-up, medication-related education, and warning signs requiring medical attention. All knowledge materials will be version-controlled and reviewed by the expert panel before being incorporated into the system. The knowledge base will be reviewed by an expert panel consisting of three gastrointestinal surgeons and two senior gastrointestinal surgical nurses before trial initiation and then updated monthly, or earlier if new clinical guidelines, departmental protocols, or safety concerns emerge. Updates will include correction of inaccurate or unclear content, addition of frequently asked patient questions, revision of safety prompts, and incorporation of newly available guideline or departmental protocol information. This system is used only for health education and self-management support and does not provide diagnosis, individualized treatment decisions, medication adjustment, or emergency medical services.

For in-scope questions, the system will provide an automated educational response based on retrieved expert-reviewed knowledge entries and predefined safety prompts. For questions beyond the scope of the knowledge base, questions with insufficient retrieval confidence, or questions involving diagnosis, treatment decisions, medication changes, emergency symptoms, or individualized clinical judgment, the system will not generate a definitive answer. Instead, it will provide a standardized safety response, reminding participants to consult their attending physician, nurse, outpatient clinic, or emergency medical service as appropriate. Examples of safety-sensitive questions include severe abdominal pain, persistent fever, active bleeding, repeated vomiting, wound dehiscence, severe dyspnea, suspected intestinal obstruction, sudden deterioration, or requests for medication adjustment. Such interactions will be recorded in the backend system, and safety-related or out-of-scope questions will be flagged for review by the research team according to the study management procedure. After each eligible query, the system may recommend related follow-up questions to encourage further learning. The system will also record backend usage data, including question categories, access frequency, and interaction duration, for subsequent research analysis and knowledge base optimization under expert supervision.

The second component is the automated health education content push module. Participants in the intervention group will receive one to three educational articles or short videos per week through the department’s official WeChat account. The content themes will follow the perioperative recovery pathway and include preoperative preparation, fasting and anesthesia-related precautions, postoperative pain management, early mobilization, respiratory training, dietary transition after gastrointestinal surgery, wound and drainage tube care, stoma care if applicable, prevention and recognition of common postoperative complications, medication and follow-up instructions, psychological adjustment, and long-term rehabilitation after discharge.

The semi-personalized content push will be implemented using a rule-based algorithm. First, all participants will receive stage-specific core content according to their perioperative phase, including the preoperative period, early postoperative hospitalization period, early post-discharge period, and 1–3 months after discharge. Second, the system will assign topic tags according to the participant’s disease type, surgical procedure, questioning history, browsing behavior, reminder settings, and expressed areas of interest. Third, the system will prioritize content according to the following order: mandatory stage-specific education, safety-related or high-frequency patient concerns, content matching the participant’s recent questions or browsing records, and general rehabilitation education. If no individualized interaction data are available, the participant will receive the default stage-based educational pathway. All pushed materials will be selected from the expert-reviewed content library, and no unreviewed educational materials will be automatically generated or pushed to participants.

The third component is the personalized reminder function, which aims to support adherence to perioperative and post-discharge self-management plans. Participants may set reminders for medication, follow-up visits, dressing changes, rehabilitation exercises, dietary tasks, or other self-management behaviors. Reminders may be set by participants themselves or with assistance from trained research nurses during system instruction. Once reminders are set, the system will automatically send notifications at the designated time. This function is intended to improve adherence to medical advice, reinforce recommended health behaviors, and support continuity of self-management after discharge.

The fourth component is the interaction and data recording mechanism. The system will automatically collect user interaction data, including platform access frequency, question types, use of educational content, reading frequency, browsing duration, and reminder use. These data will be used for intervention fidelity assessment, research analysis, and further optimization of the intervention platform. All usage data will be stored in the backend system and managed according to the study data protection procedures.

#### 2.3.2 Explanation for the choice of comparators

The control group will receive usual perioperative care and conventional health education, which is the current standard practice for patients undergoing gastrointestinal surgery in the study hospital. This comparator was chosen to determine whether adding an AI-supported health education system to routine care provides additional benefit over standard education alone. Both groups will otherwise receive the same routine medical and nursing care, discharge guidance, and follow-up management.

#### 2.3.3 Criteria for discontinuing or modifying allocated interventions

Participants may withdraw from the study at any time for any reason without any impact on their routine medical care. If the research leader believes that it is inappropriate for a subject to continue participating in the study, such as withdrawing informed consent, being unable to complete follow-up evaluations, suffering from serious illnesses, discovering serious cognitive or communication barriers after enrollment, or determining that there are other circumstances that make it inappropriate to continue participating, the research leader may also terminate the subject’s participation in the study. The data collected before withdrawal or termination of participation will be retained and analyzed as much as possible in accordance with the principle of intention to treat.

For participants assigned to the intervention group, if adverse events or safety hazards are believed to be related to misunderstandings of health information, improper self-management behavior, excessive reliance on the AI system, or system content issues, the AI-assisted health education intervention may be terminated or adjusted. In this study, excessive reliance on the AI system will be operationally defined as participant behavior suggesting that AI-generated educational information was used as a substitute for professional medical consultation or clinical advice. This may include delaying medical consultation despite warning symptoms after using the system, changing medication, diet, wound care, or rehabilitation practices without consulting healthcare professionals, repeatedly seeking advice from the AI system for safety-sensitive symptoms despite system prompts to consult clinicians, or explicitly reporting that clinical consultation was avoided because of the AI response. Such events will be identified through participant self-report, follow-up interviews, clinician or nurse reports, adverse event documentation, and backend system logs of safety-sensitive or out-of-scope questions.

In such cases, if necessary, relevant system functions will be suspended, participants will receive additional clinical guidance, and the research team will review and revise the knowledge base or system content. Serious adverse events will be recorded in accordance with research safety procedures and reported to the principal investigator and ethics committee. If safety issues indicate that the trial is no longer suitable to continue, the study may be terminated prematurely.

#### 2.3.4 Intervention delivery deviations

In this experiment, “non-compliant intervention events” refer to situations where the AI-assisted health education system is unable to deliver or operate as required by the protocol. For example, if the system is temporarily inaccessible, the reminder function is malfunctioning, the content delivery is incorrect or incomplete, the generated response exceeds the predefined expert knowledge base, or other technical or content-related issues that may affect the integrity or security of the intervention. A generated response will be considered to exceed the predefined expert knowledge base if it contains information or recommendations that cannot be traced to the expert-reviewed knowledge materials, is inconsistent with the approved knowledge base, or provides diagnosis, treatment decisions, medication adjustment, or emergency management advice beyond the educational scope of the system. Such events will be identified through backend interaction logs, participant or clinician reports, and regular manual review by the research team, and will be recorded by number, type, severity, corrective measures, and potential participant exposure.

If such an event occurs, the research team will evaluate its nature, duration, potential impact on subject safety, and potential impact on the research results. If necessary, the relevant functions of the system will be temporarily suspended, and the subjects will continue to receive routine perioperative care and traditional health education. The research team will determine whether the issue can be resolved through technical fixes, content revisions, expert reviews of the knowledge base, or temporary limitations on specific system functionalities. If the event is deemed to pose a safety risk, additional clinical guidance will be provided to participants and adverse event reporting procedures will be followed as specified in the study protocol.

All deviations in the implementation of interventions will be recorded in the study records, including the type of deviation, affected functions, duration, corrective measures, and whether any participants were exposed to potentially inappropriate information. Participants will be informed during the informed consent process and in their information materials that there may be technical or content related deviations, and such events will be handled under the supervision of the researcher. If it is determined that significant deviations have substantially affected the expected implementation of intervention measures, they will be considered in the analysis according to the plan. However, where possible, randomized subjects will still be included in the primary intention to treat analysis, and all adverse events and serious adverse events will continue to be reported regardless of adherence to the protocol.

If repeated or severe deviations in intervention implementation indicate that the AI support system can no longer be safely or consistently implemented, researchers may suggest temporarily suspending or prematurely terminating the trial, subject to review by the principal investigator and ethics committee.

#### 2.3.5 Strategies to improve adherence to interventions

Participants will receive study instructions at enrollment. Those allocated to the intervention group will additionally be instructed on how to use the AI-supported health education system through the departmental WeChat public account. Adherence to study procedures will be promoted through WeChat and telephone reminders before follow-up assessments. Engagement with the intervention will be monitored using backend usage data. If necessary, additional contact or technical guidance will be provided to support continued participation. Participants who complete the study follow-up procedures will receive a small gift as a token of appreciation. The same follow-up reminder procedures will apply to both study groups.

#### 2.3.6 Relevant concomitant care permitted or prohibited during the trial

All participants will continue to receive usual perioperative medical and nursing care during the trial. Concomitant care required for routine clinical management will be permitted and recorded as appropriate. The AI-supported system is intended for health education only and must not replace professional medical consultation or treatment. Participants in the control group will not have access to the study AI-supported health education system during the trial period.

If a participant is readmitted to hospital for any reason during the study period, the participant will remain in the originally assigned group and will not be re-randomized. The scheduled follow-up assessments will continue according to the original study timeline. The reason, timing, duration, and clinical outcome of readmission will be recorded, together with any potential relationship to the intervention. Participants in the intervention group will retain access to the AI-supported health education system when clinically appropriate. However, during readmission, acute clinical problems, treatment decisions, medication adjustment, and emergency symptoms must be managed by the attending clinical team rather than through the AI system. If the participant’s clinical condition makes continued use of the system inappropriate, the intervention may be temporarily suspended and resumed after stabilization. Readmissions suspected to be related to misunderstanding of health information, inappropriate self-management, delayed medical consultation, or system content issues will be recorded and reported as adverse events or serious adverse events according to the study safety procedures.

#### 2.3.7 Provisions for post-trial care

This is a low-risk behavioral intervention study. After trial completion, participants will continue to receive routine clinical care according to standard practice. If adverse events or serious adverse events occur during the study, appropriate clinical management will be provided, and study-related events will be handled and reported in accordance with institutional requirements and applicable regulations.

#### 2.3.8 Outcomes

The primary outcome of this trial is the change in anxiety symptoms, measured using the Hospital Anxiety and Depression Scale-Anxiety subscale (HADS-A), in patients undergoing gastrointestinal surgery who receive the AI-supported health education intervention compared with those receiving usual health education. HADS-A scores will be assessed at baseline (T0), discharge (T1), 3 months (T2), and 6 months (T3). The primary analysis will evaluate differences between groups over time.

Secondary outcomes of interest include depressive symptoms measured by the Hospital Anxiety and Depression Scale-Depression subscale (HADS-D), perceived social support measured by the Perceived Social Support Scale-Short Form (PSSS-SF), self-efficacy measured by the New General Self-Efficacy Scale-Short Form (NGES-SF), health-related quality of life measured by the EuroQol five dimensions five levels (EQ-5D-5L) including the EQ-VAS, and eHealth literacy measured by the eHealth Literacy Scale (eHEALS). These outcomes will also be assessed at baseline, discharge, 3 months, and 6 months.

In addition, safety outcomes will include adverse events and serious adverse events related to the intervention, particularly events associated with misunderstanding of health information, inappropriate self-management behavior, delayed medical consultation, or other undesirable consequences related to use of the AI-supported system. Such events will be continuously monitored throughout the study.

The exploratory research findings will include system usage data for the intervention group, such as access frequency, number and type of submitted questions, usage of educational content, reading duration, and usage of reminder functions. These data will be used to describe participants’ participation in intervention measures and explore whether system adherence is related to intervention effectiveness.

#### 2.3.9 Questionnaires

The HADS-A will be used to assess anxiety symptoms as the primary outcome (30). The Hospital Anxiety and Depression Scale consists of 14 items, including two 7-item subscales measuring anxiety (HADS-A) and depression (HADS-D). Each item is scored on a 4-point scale ranging from 0 to 3, with subscale scores ranging from 0 to 21. Higher HADS-A scores indicate more severe anxiety symptoms. Previous validation studies have shown acceptable to good internal consistency for the anxiety subscale, with Cronbach’s α values generally ranging from 0.68 to 0.93 (30–32).

The HADS-D will be used to assess depressive symptoms as a secondary outcome (30). HADS-D includes 7 items rated on the same 4-point scale as HADS-A, yielding a total score from 0 to 21. Higher scores indicate more severe depressive symptoms. Previous validation studies have shown acceptable to good internal consistency for the depression subscale, with Cronbach’s α values generally ranging from 0.67 to 0.90 (30–32).

The PSSS-SF will be used to assess participants’ perceived social support (33). This scale consists of three items that reflect different dimensions of support, including family support, friend support, and other support. Each project is scored using a 7-point Likert scale, ranging from 1 (strongly disagree) to 7 (strongly agree), with a total score range of 3–21. The higher the score, the higher the perceived level of social support. This scale has shown good internal consistency, with a Cronbach alpha coefficient of 0.84 (34).

The NGES-SF will be used to assess self-efficacy, which is defined as an individual’s confidence in their ability to complete tasks and respond to challenges. This scale consists of three items, covering perceptual ability, confidence intensity, and the universality of confidence. Each project is scored using a 5-point Likert scale, ranging from 1 (strongly disagree) to 5 (strongly agree), with a total score range of 3–15 points. The higher the score, the higher the individual’s level of self-efficacy. The scale shows good internal consistency, with a Cronbach alpha coefficient of 0.876 (35).

The EQ-5D-5L will be used to assess health-related quality of life. This instrument includes five health dimensions: mobility, self-care, usual activities, pain/discomfort, and anxiety/depression (36). Each dimension is rated on five levels, ranging from no problems to extreme problems, generating a five-dimensional health profile. In addition, the EQ-5D-5L includes the EQ visual analogue scale (EQ-VAS), on which participants rate their current overall health status on a scale from 0 to 100. Higher EQ-VAS scores indicate better self-perceived health status. In seven studies involving cancer patients, EQ-5D showed high validity and reliability, with Cronbach’s alpha coefficients ranging from 0.72 to 0.90 (37–43).

The eHEALS will be used to assess participants’ perceived ability to seek, understand, evaluate, and use electronic health information to solve health-related problems (44). The scale contains five items rated on a 5-point Likert scale from 1 (strongly disagree) to 5 (strongly agree), with total scores ranging from 5 to 25. Higher scores indicate better eHealth literacy and its Cronbach alpha coefficient of 0.913 (45).

#### 2.3.10 Participant timeline

Table 1 shows the participant timeline.

**Table 1:** The schedule of enrolment, interventions, and assessments.

Study period	Recruitment	Intervention		Follow-up
Timepoint	0	Discharge	3 Months	6 Months
Eligibility screen	√			
Informed consent	√			
Allocation	√			
Sociodemographic variables	√			
HADS-A	√	√	√	√
HADS-D	√	√	√	√
PSSS-SF	√	√	√	√
NGES-SF	√	√	√	√
EQ-5D-5L	√	√	√	√
eHEALS	√	√	√	√

eHEALS, The eHealth Literacy Scale; EQ-5D-5L, EuroQol-5 Dimensions 5 Levels; HADS-A, the Hospital Anxiety and Depression Scale-Anxiety subscale; HADS-D, the Hospital Anxiety and Depression Scale-Depression subscale; PSSS-SF, The Perceived Social Support Scale; NGES-SF, The New General Self-Efficacy Scale-Short Form.

#### 2.3.11 Sample size

The sample size was calculated based on the primary outcome, namely anxiety symptoms measured using the Hospital Anxiety and Depression Scale-Anxiety subscale. Although a gastrointestinal-surgery-specific minimal clinically important difference (MCID) for the HADS-A has not been definitively established, previous studies in medical and surgical populations have reported MCID or minimal important difference estimates for the HADS-A or HADS subscales ranging approximately from 1.2 to 4.4 points (46–49). Therefore, considering the clinical context of perioperative anxiety management and to avoid overestimating the intervention effect, we selected a conservative between-group difference of 2.0 points on the HADS-A as the target clinically meaningful effect.

The sample size for comparing two independent groups was calculated using the following formula:

n=2×(Zα/2+Zβ)2×σ2/δ2

where *δ* represents the expected between-group difference in HADS-A score, *σ* represents the assumed standard deviation, *α* represents the type I error rate, and *β* represents the type II error rate. Based on previous randomized controlled trials of perioperative educational or psychological interventions using HADS-A as an outcome measure, the standard deviation was assumed to be 3.4 (50, 51). With a two-sided α of 0.05, 80% power, *δ* = 2.0, and *σ* = 3.4, the required sample size was calculated as 45.3 participants per group.

Therefore, at least 46 participants were required in each group. Considering that this trial includes a 3-month intervention and follow-up assessments up to 6 months, an attrition rate of approximately 20% was anticipated due to postoperative recovery, possible readmission or transfer, reduced willingness to complete follow-up questionnaires, and loss to follow-up. After adjustment for attrition, the required sample size was 46 / (1–0.20) = 57.5 participants per group, which was rounded up to 60 participants per group. Assuming an attrition rate of no more than 20%, approximately 48 participants per group are expected to remain available for analysis, which remains above the minimum required sample size of 46 participants per group. Although anxiety may naturally decrease after discharge in both groups, the primary analysis will focus on the between-group difference in HADS-A change over time using a repeated-measures linear mixed-effects model. Therefore, the sample size was considered sufficient to detect the prespecified 2.0-point between-group difference under the stated assumptions. A total of 120 participants will be recruited for this trial.

#### 2.3.12 Recruitment

The participants will be recruited from the Department of Gastroenterology Surgery at Chengdu Third People’s Hospital. Potential participants will be identified during hospitalization and evaluated based on pre-defined inclusion criteria. The recruitment process will focus on continuous recruitment of hospitalized patients in the department who meet the research criteria and provide written informed consent.

### 2.4 Assignment of interventions: allocation

#### 2.4.1 Sequence generation

Eligible participants will be randomly assigned to either the intervention group or the control group in a 1:1 ratio. The random allocation sequence will be generated before recruitment by an independent statistician who will not be involved in participant recruitment, intervention delivery, outcome assessment, or data analysis. The allocation list will be generated using R statistical software (R Foundation for Statistical Computing, Vienna, Austria) with a reproducible random seed (52). A computer-generated random permutation of group assignments will be created according to the 1:1 allocation ratio, and each allocation code will correspond to a unique sequential study number.

#### 2.4.2 Concealment mechanism

Allocation concealment will be implemented using sequentially numbered, opaque, sealed envelopes. After the randomization sequence has been generated, the group assignments will be placed into identical opaque envelopes according to the sequential study numbers. The envelopes will be sealed, signed across the seal, and kept by an independent research coordinator who will not participate in participant recruitment or intervention delivery. The allocation sequence will remain concealed from the investigators responsible for screening and enrolling participants until eligibility has been confirmed and written informed consent has been obtained. The next envelope in sequence will then be opened to determine the participant’s group assignment. As this is an open-label trial, the assigned group will be disclosed to the participant and relevant research staff only after allocation has been completed.

#### 2.4.3 Implementation

The randomization process will involve separated roles to reduce selection bias. The independent statistician will generate the random sequence, the independent research coordinator will prepare and keep the sealed envelopes, and the recruiting investigator will screen participants, obtain written informed consent, assign the next sequential study number, and open the corresponding envelope only after enrollment. Participants will then receive the study procedures corresponding to their allocated group.

To reduce contamination between groups, participants allocated to different groups will be managed in separate ward areas after enrollment whenever ward conditions allow. In the study department, inpatient beds are distributed across two ward floors. Therefore, participants in the intervention and control groups will be preferentially arranged on different floors according to their randomized allocation. Bed assignment will be coordinated with ward nursing staff after randomization, and participants from the two groups will not be placed in the same room when feasible.

In routine clinical practice, complete physical separation may not always be possible because of bed availability, patient transfer, clinical urgency, infection control requirements, or other ward management needs. Therefore, any deviation from the planned separation strategy, including placement on the same floor or room transfer between ward areas, will be recorded in the study log. Health education sessions, WeChat-based intervention instructions, QR codes, and intervention-related materials will be provided only to participants in the intervention group. Participants will also be reminded not to share intervention materials or AI system access with other patients. Potential contamination will be monitored through ward and bed assignment records, intervention material distribution records, backend AI-system access logs, and participant self-report during follow-up. Control group participants will receive usual perioperative care and conventional health education only.

### 2.5 Assignment of interventions: blinding

#### 2.5.1 Who will be blinded

This is an open-label trial. Participants and research staff implementing the intervention will not be blinded because of the nature of the study intervention, namely access to an AI-supported health education system embedded in a WeChat public account. To reduce assessment bias, outcome assessors and statisticians will remain blinded to group allocation until database lock.

#### 2.5.2 Procedure for unblinding if needed

As the trial is open label, there is no formal unblinding procedure for participants or intervention staff. Outcome assessors and statisticians will be unblinded only after completion of data collection and database lock.

### 2.6 Data collection and management

#### 2.6.1 Plans for assessment and collection of outcomes

Study data will be collected using structured questionnaires, hospital medical records, and backend data automatically recorded by the AI-supported health education system. Baseline data will be collected after enrollment and before randomization, including sociodemographic characteristics, disease-related information, and baseline outcome measures. Outcome assessments will be performed at baseline (T0), discharge (T1), 3 months (T2), and 6 months (T3).

Patient-reported outcomes, including HADS, PSSS-SF, NGES-SF, EQ-5D-5L, and eHEALS, will be collected using standardized questionnaires. During hospitalization, participants will be guided by the research team to complete the baseline and discharge assessments. During the intervention and follow-up periods, outcome data will be collected mainly through electronic questionnaires distributed via the study WeChat public account, supplemented by telephone follow-up and outpatient follow-up where applicable. For participants who are unable to complete online questionnaires independently, trained research staff will administer the questionnaires by telephone and record the responses item by item to maximize data completeness.

For participants in the intervention group, the AI-supported system will automatically record usage data, including question frequency and categories, login frequency, duration of use, educational content access, reading duration, and reminder function use. These data will be used to assess participant engagement with the intervention and to support exploratory analyses of adherence and usage patterns. Additional clinical information, including diagnosis, treatment-related variables, follow-up status, and adverse events, will be obtained from hospital records and study follow-up documentation.

All quantitative data will be entered into a unified electronic study database using standardized data collection forms. The questionnaires and data collection procedures will be pilot-tested before formal study initiation to ensure clarity and feasibility.

#### 2.6.2 Plans to promote participant retention and complete follow-up

Participants will receive detailed information about the study procedures, follow-up schedule, and assessment requirements during enrollment. The importance of completing follow-up assessments will be emphasized by the research team. Participants may withdraw from the study at any time without giving a reason and without any consequences for their routine medical care. Whenever possible, participants who discontinue the intervention or withdraw early will be asked whether they are willing to complete outcome assessments already scheduled in the study.

To improve the retention rate of participants, subsequent evaluations will be conducted in a flexible manner, including sending electronic questionnaires through official WeChat accounts, conducting telephone follow-up, and conducting outpatient follow-up where applicable. This enables participants to complete the research questionnaire in a suitable format at a convenient time. Prior to each planned follow-up, participants will receive reminder messages via WeChat or phone. If the questionnaire is not completed within the expected time, the research team will contact the participants again within 3–5 days to encourage them to complete the questionnaire and minimize data loss.

For participants in the intervention group, backend usage data may also help identify interruption of engagement, allowing the research team to provide additional contact or technical guidance where appropriate. In addition, participants who complete the study follow-up procedures will receive a small gift as a token of appreciation. The value of this gift will be modest so as not to unduly influence participation.

#### 2.6.3 Data management

All study data will be entered into a unified electronic study database using standardized data collection forms. Quantitative data will be managed using a double-entry process, and logical checks and consistency verification will be performed to ensure data accuracy. Source data will include structured questionnaires, hospital medical records, study follow-up records, and backend data automatically recorded by the AI-supported health education system. Adverse events and serious adverse events will be documented in the study database throughout the trial.

All original questionnaire materials will be assigned study identification numbers and stored centrally. Electronic research data will be stored in encrypted form with role-based access control, and access will be limited to authorized study personnel. Identifiable participant information will be stored separately from research data, and a unique study code will be used in place of directly identifying information whenever possible to protect confidentiality. Written informed consent forms and any paper records will be stored securely in a restricted-access location within the hospital.

During the study, regular data quality reviews will be performed, including checks for missing values, outliers, and data completeness. Any corrections or updates to the database will be documented in an auditable manner. At the end of the study, the database will be locked, and no further changes will be made without authorization from the principal investigator. Data storage, retention, and archiving will be conducted in accordance with institutional requirements and applicable regulations.

#### 2.6.4 Confidentiality

All research data will be recorded and stored using a unique study identification code for each participant. Identifiable personal information will be kept separately from research data, and only authorized members of the research team will have access to the identification code list. The code key will be securely maintained by the principal investigator in accordance with institutional research management requirements.

Electronic study data will be stored in encrypted databases with access restricted to authorized study personnel. Paper documents, including signed informed consent forms, will be stored in a secure location within the hospital with restricted access. All data collected from questionnaires, hospital records, follow-up procedures, and backend system logs will be handled in a de-identified manner whenever possible. No directly identifiable participant information will be disclosed in presentations, reports, or publications arising from this study.

#### 2.6.5 Plans for collection, laboratory evaluation, and storage of biological specimens for genetic or molecular analysis in this trial/future use

No biological specimens will be collected, stored, or analyzed in this trial. This study is a behavioral and digital health intervention study and does not involve laboratory evaluation, genetic analysis, or future use of biological materials.

### 2.7 Statistical methods

#### 2.7.1 Statistical methods for primary and secondary outcomes

All statistical analyses will be performed using appropriate statistical software. The primary analysis will follow the intention-to-treat (ITT) principle, including all randomized participants in the groups to which they were originally assigned, regardless of adherence to the allocated intervention. A per-protocol (PP) analysis will also be conducted as a sensitivity analysis to assess the robustness of the findings.

Descriptive summary of baseline characteristics will be conducted by research group. Continuous variables with an approximate normal distribution will be represented as mean ± standard deviation, while variables with a non normal distribution will be summarized as median and interquartile range. The categorical variables will be expressed in frequency and percentage. In appropriate circumstances, comparisons of baseline characteristics between groups can be described using independent sample *t*-tests or Mann Whitney *U* tests (for continuous variables), as well as chi square tests or Fisher’s exact tests (for categorical variables).

The primary outcome, anxiety symptoms measured by the HADS-A, will be analyzed using a linear mixed-effects model for repeated measures. The model will include study group, time point, and the group-by-time interaction as fixed effects, with participant included as a random effect to account for within-subject correlation across repeated assessments. The main treatment effect will be evaluated by the group-by-time interaction, which reflects whether changes in HADS-A over time differ between the intervention and control groups. Estimated marginal means, mean differences between groups at each time point, 95% confidence intervals, and corresponding *p* values will be reported.

The secondary outcomes, including depressive symptoms (HADS-D), perceived social support (PSSS-SF), self-efficacy (NGES-SF), health-related quality of life (EQ-5D-5L, including EQ-VAS), and eHealth literacy (eHEALS), will be analyzed using the same repeated-measures linear mixed-effects modeling approach. For each outcome, the effects of group, time, and group-by-time interaction will be estimated. These analyses will allow assessment of both between-group differences and changes over time.

In supplementary analysis, paired *t*-tests or Wilcoxon signed rank tests can be used to explore changes over time within the group based on data distribution. These analysis results will serve as a supplement to the main mixed model results and should be interpreted with caution. Statistical significance will be evaluated using a two-sided test, with a significance level set at 0.05.

#### 2.7.2 Interim analyses

No interim analyses are planned for this trial. Given the relatively small sample size, the low-risk behavioral nature of the intervention, and the planned follow-up duration, outcome analyses will be conducted after completion of data collection and database lock.

#### 2.7.3 Methods for additional analyses

Exploratory additional analyses are planned. In particular, descriptive and exploratory analyses will be performed using backend system data from the intervention group, including question frequency, usage frequency, reading duration, and reminder use, to examine patterns of intervention engagement. In addition, age-stratified analyses may be conducted to explore potential heterogeneity in intervention effects across different age groups. These analyses will be considered exploratory and hypothesis-generating in nature.

#### 2.7.4 Methods in analysis to handle protocol non-adherence and any statistical methods to handle missing data

The primary analysis will be conducted according to the ITT principle, including all randomized participants in the groups to which they were originally assigned. A PP analysis will also be performed as a sensitivity analysis to evaluate the robustness of the findings under conditions of better adherence to the study protocol.

Through the above-mentioned retention and follow-up procedures (including WeChat reminders, phone contacts, and flexible questionnaire filling methods), missing data will be minimized to the greatest extent possible. For primary and secondary repeated measures of outcome indicators, a linear mixed effects model will be used; Under the assumption that ‘missing data is randomly missing, these models are able to handle incomplete repeated measurement outcome data properly without the need for a complete case analysis. In addition, if necessary, multiple imputation will be used to address missing data under the missing-at-random assumption. Sensitivity analyses, such as comparison of complete-case results and imputed-data results, will be performed where appropriate to assess the stability of the findings. Loss to follow-up rates and dropout patterns between groups will also be compared.

#### 2.7.5 Plans to give access to the full protocol, participant-level data, and statistical code

The full study protocol may be made available upon reasonable request to the corresponding author, subject to institutional and ethical requirements. De-identified participant-level data and statistical code may also be made available upon reasonable request after completion of the study, provided that such sharing is consistent with applicable data protection requirements, institutional policies, and research collaboration agreements.

### 2.8 Oversight and monitoring

#### 2.8.1 Composition of the coordinating center and trial steering committee

This is a single-center study designed, conducted, and coordinated at Chengdu Third People’s Hospital. Day-to-day trial support will be provided by the study team under the supervision of the principal investigator. The principal investigator will be responsible for overall trial oversight, study conduct, participant safety, and final decisions regarding protocol implementation. Members of the research team will support participant screening and recruitment, informed consent, intervention implementation, follow-up assessments, data collection, and study documentation. Data collection and database management will be undertaken by the study team in accordance with the protocol-defined data management procedures. The AI-supported intervention system will be maintained collaboratively by the clinical and technical personnel involved in the study.

The study team includes investigators from Chengdu Third People’s Hospital and collaborating institutions, with responsibilities covering study design, project implementation, and manuscript preparation. The team will communicate regularly during the study to review recruitment progress, follow-up completion, data quality, protocol adherence, and safety issues. No separate trial steering committee, stakeholder advisory group, or public involvement group has been established for this trial.

#### 2.8.2 Composition of the data monitoring committee, its role, and reporting structure

This study will not establish a formal independent data monitoring committee (DMC). The decision was made based on the single center design used in this study, the relatively small sample size, the intervention measures being low-risk behavioral interventions, and no mid-term analysis planned.

Throughout the entire trial period, the main researcher and research team will be responsible for conducting safety monitoring. Adverse events (AEs) and serious adverse events (SAEs) will be recorded, reviewed, and managed in accordance with the study safety procedures. If a serious adverse event occurs, it will be reported immediately to the principal investigator within the prescribed reporting time limit and, if necessary, to the ethics committee. For events suspected to be related to misunderstandings of health information, improper self-management behavior, or system content issues, the relevant intervention functions can be suspended, and the knowledge base or system content can be reviewed and revised as needed. If it is believed that there are significant safety hazards, researchers may suggest temporarily suspending or terminating the experiment in advance.

#### 2.8.3 Adverse event reporting and harms

All AEs reported by participants or identified by the investigators during the study will be recorded in the study database. The severity, timing, outcome, and assessed relationship to the study intervention will be documented. In this trial, particular attention will be paid to events potentially related to the AI-supported health education intervention, including misunderstanding of health information, increased anxiety or psychological distress, inappropriate self-management behavior, delayed medical consultation, or other undesirable consequences associated with use of the system.

SAEs include events that result in death, life-threatening situations, long-term hospitalization or readmission, persistent or significant disability, and events that researchers consider to have other significant medical implications. In this study, events such as delayed treatment or worsening of the condition caused by misinterpretation of health information, which result in substantial clinical harm, can also be considered serious adverse events. Once AEs or SAEs are detected, subjects will undergo appropriate clinical evaluation and management. If there is suspicion that the event is related to the implementation of system content or intervention measures, the relevant system functions may be suspended and the knowledge base or content may be reviewed and revised as necessary.

All AEs will be recorded within 24 h after the research team becomes aware and reviewed by the principal investigator. SAEs will be reported immediately to the principal investigator and, if necessary, to the ethics committee in accordance with applicable institutional requirements and reporting deadlines. Researchers will conduct a causal relationship assessment and classify adverse events as “definitely related,” “possibly related,” “possibly unrelated,” or “unrelated to intervention.” At the end of the study, all safety data will be summarized in the final analysis.

#### 2.8.4 Frequency and plans for auditing trial conduct

Given that this study adopts a single center design and the research behavior risk is low, an independent audit institution has not been formally established. The implementation of the experiment will be supervised by the principal investigator and research team through regular reviews of recruitment status, informed consent documents, protocol compliance, follow-up completion status, data quality, and safety reports.

During the research period, regular data quality checks and internal review procedures will be conducted, including verifying the eligibility of subjects for enrollment, the integrity of informed consent documents, the integrity of source data, missing data, and the recording of adverse events and serious adverse events. Any significant protocol deviations or data quality issues identified during these review processes will be documented and addressed by the research team. In addition, according to applicable requirements, this study may also be subject to review or inspection by institutional ethics committees or other relevant regulatory agencies.

#### 2.8.5 Plans for communicating important protocol amendments to relevant parties

A substantial protocol amendment will be defined as any change to the protocol, informed consent materials, intervention procedures, outcome assessment, safety monitoring procedures, or other study documentation that may significantly affect participant safety, participant rights, the scientific validity of the trial, the conduct or management of the study, or the quality and safety of the intervention.

All major modifications must be submitted to the ethics committee and other relevant agencies for review and approval in accordance with applicable requirements before implementation, unless immediate action is required to ensure the safety of the subjects. Non-substantial amendments will be documented, dated, and retained in the trial master documentation. Study registration records and other relevant study documents will be updated as appropriate.

Participants will be promptly notified of any changes that affect them in any way, including modifications to the study protocol, follow-up requirements, risks, or data collection methods. If necessary, a revised written informed consent form will be obtained and documented before continued participation.

#### 2.8.6 Dissemination plans

The results of this study will be disseminated through academic publication and other appropriate scholarly channels after study completion. Both positive and negative findings will be reported. Study findings may be submitted to peer-reviewed journals and presented at relevant academic or professional meetings.

Dissemination of results will be conducted in a way that protects participant confidentiality, and no directly identifiable personal information will be disclosed. Where appropriate, study results may also be summarized in a form suitable for participants or the public.

## 3 Discussion

The aim of this experiment is to evaluate whether an AI health education system based on WeChat and constrained by expert knowledge can improve anxiety symptoms and other patient reported outcomes in patients undergoing gastrointestinal surgery. This study addresses an important clinical need, as perioperative education remains essential but is frequently limited by time constraints, clinical workload, and insufficient continuity of information support after discharge (53, 54). By embedding AI assisted education into widely used digital platforms, this intervention aims to provide convenient, timely, and interactive support, while leveraging expert curated knowledge bases to ensure safety.

This study has multiple advantages, including the use of a randomized controlled design, the use of a real-world and scalable delivery platform, and the adoption of a knowledge constrained AI framework aimed at reducing risks associated with unrestricted general models. At the same time, it should also be acknowledged that there are several limitations to it. As a single center, open label trial, the generalizability of its research results may be limited and may be influenced by local diagnosis and treatment patterns. In addition, participation in the study requires the use of a smartphone and access to WeChat, which may exclude some patients with low digital literacy. In addition, because the intervention is a multicomponent system that integrates AI-supported question answering, educational content delivery, reminders, and usage data recording, the present two-arm design cannot isolate the independent effect of the AI algorithm itself from the broader digital delivery model. Although participants in different groups will be preferentially managed on separate ward floors to reduce contamination, complete prevention of contamination may be difficult in a real-world hospital setting because of bed constraints, patient transfer, and informal communication among patients or caregivers. Despite these limitations, this study may still provide valuable evidence for the feasibility and effectiveness of AI assisted perioperative education in gastrointestinal surgery, and serve as a reference for developing safe and scalable digital education interventions in clinical nursing in the future.
